# The role of exercise-related FNDC5/irisin in depression

**DOI:** 10.3389/fphar.2024.1461995

**Published:** 2024-10-17

**Authors:** Yaqi Liu, Xiying Fu, Xing Zhao, Ranji Cui, Wei Yang

**Affiliations:** ^1^ Department of Neurology, The Second Hospital of Jilin University, Changchun, Jilin, China; ^2^ Jilin Provincial Key Laboratory on Molecular and Chemical Genetics, The Second Hospital of Jilin University, Changchun, Jilin, China; ^3^ Department of Endocrinology, The Second Hospital of Jilin University, Changchun, Jilin, China

**Keywords:** exercise, FNDC5/irisin, depression, metabolism, BDNF, neurogenesis, inflammation, oxidative stress

## Abstract

The complexity of depression presents a significant challenge to traditional treatment methods, such as medication and psychotherapy. Recent studies have shown that exercise can effectively reduce depressive symptoms, offering a new alternative for treating depression. However, some depressed patients are unable to engage in regular physical activity due to age, physical limitations, and other factors. Therefore, pharmacological agents that mimic the effects of exercise become a potential treatment option. A newly discovered myokine, irisin, which is produced during exercise via cleavage of its precursor protein fibronectin type III domain-containing protein 5 (FNDC5), plays a key role in regulating energy metabolism, promoting adipose tissue browning, and improving insulin resistance. Importantly, FNDC5 can promote neural stem cell differentiation, enhance neuroplasticity, and improve mood and cognitive function. This review systematically reviews the mechanisms of action of exercise in the treatment of depression, outlines the physiology of exercise-related irisin, explores possible mechanisms of irisin’s antidepressant effects. The aim of this review is to encourage future research and clinical applications of irisin in the prevention and treatment of depression.

## 1 Introduction

Depression is a common and serious mental illness that affects approximately 300 million people worldwide and is the third leading cause of the global illness burden (Herrman et al., 2019). It is also one of the leading disabling conditions globally, accounting for nearly 47 million disability cases in 2019, and is expected to become the top global health problem by 2030 (17 Oct 2020, 2020; Friedrich, 2017). Depression is a disease involving genetic, psychological, biochemical, and socio-environmental factors, resulting in severe physical, psychological, and economic burdens for patients and their families (Hammen, 2018). Consequently, identifying effective measures for the prevention and treatment of depression has significant implications for improving population health. Clinical data indicate that current treatments for depression have various limitations regarding their onset of action time and overall efficacy (Wang et al., 2015). Traditional antidepressants often takes weeks or even months to alleviate depressive symptoms, has multiple side effects, and is ineffective for approximately one-third of patients, limiting treatment efficacy (Cipriani et al., 2018). Therefore, further research into the pathophysiology of depression is essential for exploring new therapeutic targets.

Regular exercise not only helps with skeletal muscle growth and development, but also improves brain function and broadly enhances physical and mental health throughout the lifespan (Schulkin, 2016). Several studies have demonstrated that exercise positively impacts cognitive function, memory, and mental health in both human and animal models (Wong-Goodrich et al., 2010; Pietropaolo et al., 2008). Exercise has been recommended as an enhancement strategy for the treatment of depression (Lee et al., 2021; Trivedi et al., 2011). More than 40 meta-analyses and other systematic reviews have confirmed the effectiveness of exercise in alleviating depressive symptoms. Research has identified exerkines as key contributors to the positive effects of exercise (Safdar et al., 2016). Coined by Tarnopolsky et al., this term refers to cytokines, humoral factors, and metabolites produced during exercise that act in a paracrine or endocrine manner, contributing to the systemic benefits of exercise (Safdar and Tarnopolsky, 2018).

During physical activity, skeletal muscle releases different myokines, some of which can cross the blood-brain barrier (BBB) and mediate various beneficial effects associated with exercise. Irisin, a myokine produced during exercise through the cleavage of its precursor protein, fibronectin type III domain-containing protein 5 (FNDC5). Irisin promotes energy metabolism, improves insulin resistance, regulates disorders of glucose and lipid metabolism, and is associated with browning of adipose tissue for thermogenesis (Boström et al., 2012; Lee et al., 2014). Recent research suggests that irisin plays a beneficial role in neurological disorders by regulating energy metabolism, enhancing synaptic plasticity, fostering neurogenesis, reducing neuroinflammation, and inhibiting oxidative stress (Islam et al., 2021; Lourenco et al., 2019; Wang et al., 2022; Zhang et al., 2016). As a novel exercise-induced myokine, irisin serves as a crucial communication link between the skeletal muscle and the brain.

Preliminary studies indicate that irisin may exert antidepressant effects and could be a potential target for depression treatment (Wang and Pan, 2016; Siteneski et al., 2018; Pignataro et al., 2022). We conducted a thorough search of PubMed publications from 2012 to 2024 using the following keywords: exercise, FNDC5, irisin, depression, central nervous system, and neuroprotection. On this basis, we describe the positive effects of exercise on the brain and its preventive and therapeutic potential for depression, highlight the relationship between exercise-associated irisin and depression, and further explore the potential therapeutic role of FNDC5/irisin in depression and its possible mechanisms.

## 2 The role of exercise in the prevention and treatment of depression

Exercise has long been recognized for its benefits to overall health, and regular physical activity has been associated with better physical health outcomes (Warburton et al., 2006). Consistent evidence supports the effectiveness of exercise-based interventions in reducing the incidence of both physical and mental illnesses and in lowering all-cause mortality rates (Chekroud et al., 2018). Exercise has been shown to be an effective treatment for mild to severe depression, with response rates comparable to those of commonly used treatments, such as antidepressant medications and cognitive behavioral therapy (Babyak et al., 2000; Blumenthal et al., 2007). Moreover, exercise as an adjunct to standard antidepressant therapy has demonstrated significant potential for treating severe depression (Gourgouvelis et al., 2017). In fact, the combination of exercise with standard treatment has been found to produce significantly greater antidepressant effects than standard treatment alone (Lee et al., 2021). Exercise also plays a preventive role, reducing the risk of depression onset (Mammen and Faulkner, 2013). Numerous clinical studies have explored the link between depression and exercise. A recent systematic review and meta-analysis of 15 prospective studies, encompassing over two million person-years, found that physical activity was inversely associated with the development of depression, with the most significant risk reduction seen at lower levels of physical activity (Pearce et al., 2022). Another meta-analysis of 49 prospective cohort studies, including 1,837,794 person-years, conducted by Schuch et al., showed that individuals engaging in high - intensity exercise were less likely to develop depression compared with those leading a sedentary lifestyle. This protective effect of physical activity against depression was observed regardless of age, gender, or geographic region (Schuch et al., 2018).

**FIGURE 1:**
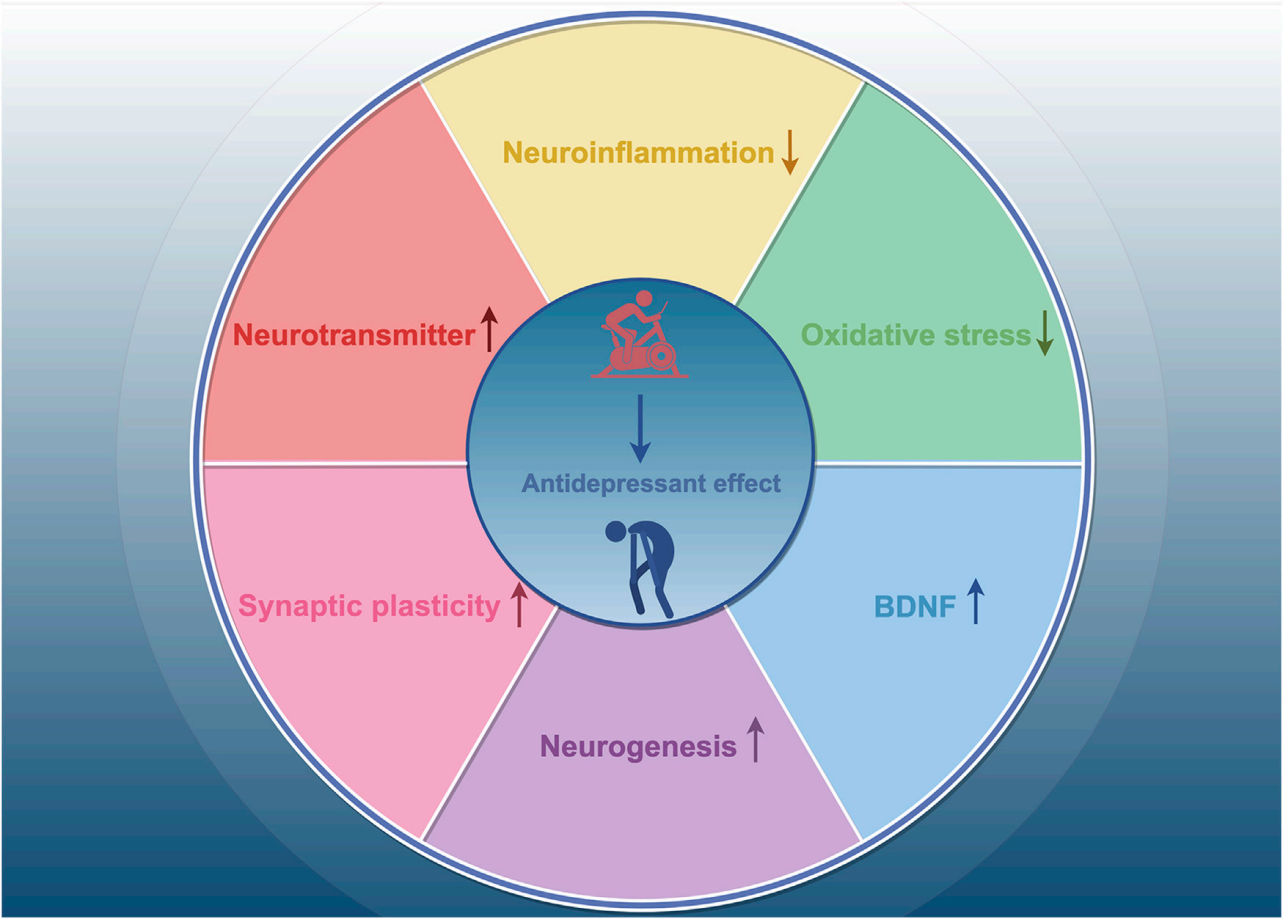
The mechanisms of exercise’s antidepressant effects. Exercise exerts antidepressant effects by increasing neurotransmitter levels, decreasing neuroinflammation, inhibiting oxidative stress, and enhancing synaptic plasticity, neurogenesis, and BDNF levels.

We have synthesized findings from rodent and human studies to summarize the neurobiological mechanisms underlying the antidepressant effects of exercise. Exercise has been shown to influence neurotransmitters such as dopamine (DA), norepinephrine (NE), and 5-hydroxytryptamine (5-HT) (Lin and Kuo, 2013; Cunha et al., 2013). In a randomized controlled trial, patients with depression who engaged in 16 weeks of physical activity exhibited increased plasma levels of the anti-inflammatory cytokine interleukin-10 (IL-10), along with decreased levels of pro-inflammatory cytokines and pro-inflammatory markers, such as C-reactive protein and interleukin-6 (IL-6), and reduced neutrophil and monocyte counts (Euteneuer et al., 2017). Animal study has also shown that exercise reduces depression-like behaviors, which are associated with changes in systemic inflammation, such as increased IL-10 levels (Sigwalt et al., 2011). These findings suggest that regular physical activity have anti-inflammatory effects. Oxidative stress-related endothelium damage has been linked to the onset and progression of several disorders, including vascular depression and late-life depression (Luca and Luca, 2019). Brocardo et al. found significant increases in lipid peroxidation and protein oxidation in the hippocampus and cerebellum of rats exposed to ethanol; in contrast, voluntary running wheel exercise raised endogenous levels of the antioxidant glutathione and alleviated depression-like behaviors in male rats (Brocardo et al., 2012). Recent research suggests that the antidepressant effects of exercise are associated with improvements in synaptic plasticity, neuronal growth, and neurogenesis in the adult hippocampus (Liang et al., 2022; Liang et al., 2019; Micheli et al., 2018; Steib et al., 2014). In a mouse model of chronic unpredictable stress (CUS)-induced depression, Liang et al. discovered that exercise increased the number of mature neurons and dendritic spines in the hippocampal CA1, CA3, and dentate gyrus (DG) regions and was more effective than fluoxetine in promoting neuronal maturation and modulating synaptic plasticity (Liang et al., 2022). A growing body of evidence suggests that brain-derived neurotrophic factor (BDNF) mediates the antidepressant effects of exercise. Clinical trials have shown that acute exercise increases BDNF levels in an intensity-dependent manner, and changes in BDNF are strongly associated with improvements in depressed mood following exercise (Meyer et al., 2016). In animal studies, 28 consecutive days of physical activity elevated BDNF mRNA and protein expression in hippocampal neurons and specifically increased BDNF levels in the dendrites of CA3 neurons (Baj et al., 2012). Collectively, these findings suggest that neurotransmitters, neurotrophic factors, neuroinflammation, oxidative stress, neurogenesis, and neuroplasticity are involved in the antidepressant mechanisms of exercise (Figure 1).

## 3 Source and biological function of irisin

**FIGURE 2:**
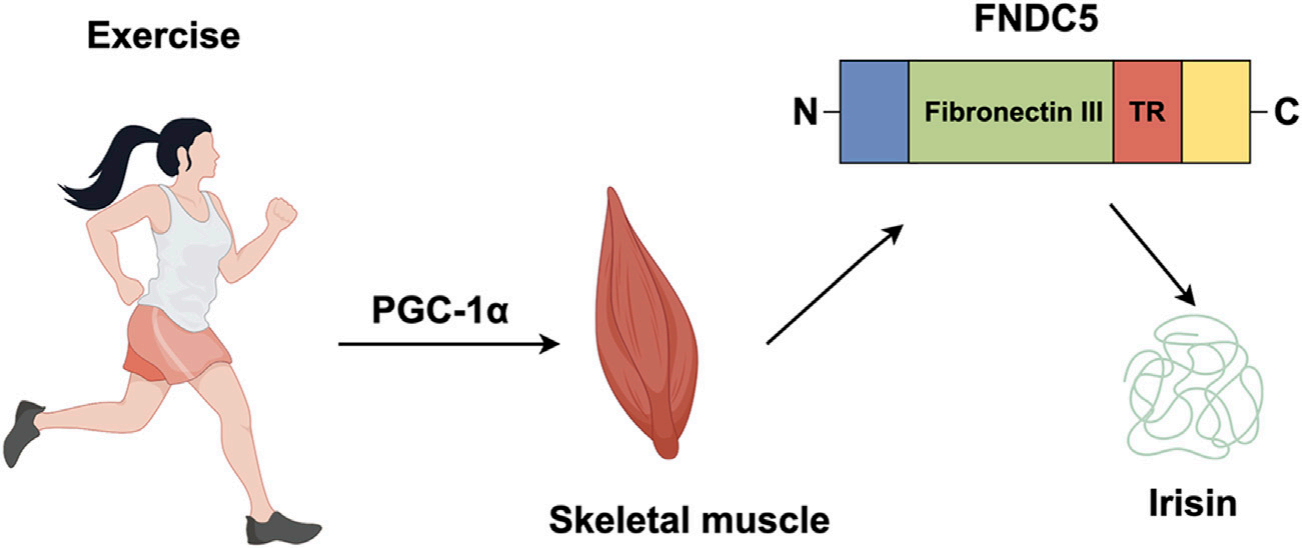
Irisin is produced by exercise. In response to exercise peroxisome proliferator, PGC-1α, leads to the production of FNDC5, a protein which contains an N-terminal signal sequence (blue); fibronectin III (green); a transmembrane region (TR) (red); a cytosolic region with a C-terminal tail (yellow). The proteolytic cleavage of mature FNDC5 results in the release of irisin.

Irisin, a myokine released into the circulation during exercise, was first identified by Boström and colleagues in 2012 (Boström et al., 2012). It is cleaved from FNDC5, a transmembrane precursor protein whose transcription is regulated by peroxisome proliferator-activated receptor gamma coactivator 1-alpha (PGC-1α), a key transcriptional cofactor involved in energy metabolism (Boström et al., 2012). Exercise-induced skeletal muscle contraction stimulates, which directly upregulates FNDC5 expression, leading to irisin synthesis and secretion. FDNC5 consists primarily of a signal peptide (amino acids 1–31) and a fibronectin III structural domain (amino acids 32–212) that contains irisin (amino acids 32–143), with the remaining amino acids forming transmembrane and cytoplasmic domain (Figure 2) (Boström et al., 2012; Nie et al., 2020; Schumacher et al., 2013).

Irisin is predominantly produced by skeletal and cardiac muscles, with the latter producing higher levels (Boström et al., 2012; Huh et al., 2012). However, its production is not confined to muscle tissue; other organs, including the liver, thyroid, adrenal glands, bladder, ovaries, and CNS, also express FNDC5 and release irisin (Huh et al., 2012; Dun et al., 2013; Aydin et al., 2014; Ruan et al., 2019). Emerging research shows that irisin is widely expressed in the brain, particularly in Purkinje cells of the cerebellum and in the cerebral cortex, hippocampus, putamen, and hypothalamus. It plays a crucial role in normal physiological processes in the brain, and is linked to neurodegenerative diseases (Dun et al., 2013). It is unclear, whether cerebrospinal fluid irisin originates from peripheral irisin or central FNDC5. Ruan et al. proposed that irisin can cross the BBB through a saturable transport system (Ruan et al., 2019). Supporting this, peripheral FNDC5 gene overexpression has been shown to influence hippocampal gene expression and mitigate memory impairment (Islam et al., 2021; Lourenco et al., 2019). Irisin’s receptors are the αV integrin family, specifically the αV/β5 integrin complex. Inhibition of αV integrins in osteocytes and adipocytes suppresses irisin’s signaling and activity (Kim et al., 2018). Notably, integrin αVβ5 is highly expressed in microglia (Welser-Alves et al., 2011). Wang et al. demonstrated that irisin can reduce neuroinflammation after cerebral hemorrhage by binding to integrin αVβ5 expressed in microglia (Wang et al., 2022).

Irisin is considered a critical mediator between exercise and metabolic homeostasis. It enhances the expression of uncoupling protein 1 (UCP1), which promotes the conversion of white adipose tissue (WAT) to brown adipose tissue (BAT), generating heat. Additionally, irisin regulates glucose uptake and mitochondrial biogenesis (Boström et al., 2012; Lee et al., 2014). Numerous studies have confirmed its beneficial effects in the development of various diseases, including obesity, type 2 diabetes (T2D), osteoarthritis, cardiovascular diseases, ischemic stroke, intracerebral hemorrhage, and neurodegenerative diseases like Alzheimer’s and Parkinson’s disease (Islam et al., 2021; Wang et al., 2022; Li et al., 2017; Askari et al., 2018; Kam et al., 2022; Yan et al., 2022). FNDC5/irisin activates several intracellular signaling pathways to exert its biological effects. The mitogen-activated protein kinase (MAPK) pathway is a key mechanism in white adipocyte browning, neural differentiation, and osteoblast proliferation. Other pathways, such as AMP-activated protein kinase (AMPK), phosphoinositide 3-kinase/protein kinase B (AKT), cAMP/protein kinase A (PKA)/cAMP response element-binding protein (CREB), and signal transducer and activator of transcription 3 (STAT3)/Snail, are involved in other crucial FNDC5/irisin functions (Lourenco et al., 2019; Wang et al., 2022; Rabiee et al., 2020).

## 4 FNDC5/irisin and exercise

Exercise is an essential factor in inducing irisin secretion from skeletal muscle. Tandem mass spectrometry can be used to identify and quantify circulating irisin in humans. Sedentary individuals have irisin levels around 3.6 ng/mL, which rise significantly to 4.3 ng/mL after aerobic interval training (Jedrychowski et al., 2015). Flori et al. reveal that other factors such as temperature, diet, and certain medicines like fenofibrate and metformin also affect irisin levels (Flori et al., 2021). As summarized in Table 1, numerous human and animal studies have demonstrated that exercise increases irisin levels in the bloodstream and hippocampus and elevates FNDC5 mRNA and protein expression in muscle tissue (Boström et al., 2012; Brenmoehl et al., 2014; Pang et al., 2018; Norheim et al., 2014; Babaei et al., 2021). Resistance, aerobic or combined exercise seems to play a positive role. Notably, the level of irisin produced corresponds to an increase in exercise intensity (Tsou et al., 2019). However, in several studies, exercise did not lead to changes in serum irisin (Pardo et al., 2014). Hecksteden et al. (2013) found that neither chronic endurance nor resistance exercise could increase circulating irisin. Several factors need to be considered in clinical trials, such as the age, gender, weight, and fitness level of the subjects. The most critical factors are the timing of sample collection and the type of exercise regimen used, which may lead to the failure of some studies to find an association between exercise and irisin secretion (Tsuchiya et al., 2015; He et al., 2018; Ruas et al., 2012). Since the half-life of irisin in the body is less than 1 h, it is necessary to pay attention to the time of blood collection after a single exercise intervention (Jedrychowski et al., 2015). Therefore, precision studies of irisin following exercise intervention needs to be further clarified.

**TABLE 1:** Changes in FNDC5 and/or irisin levels after exercise.

Species	Motion type	Level change	References
C57BL/6J mice	Treadmill exercise	Irisin (plasma) ↑ FNDC5 mRNA and protein (muscle) ↑	Pang et al. (2018)
Wistar rats	Treadmill exercise	FNDC5 protein (hippocampal) ↑	Babaei et al. (2021)
Outbred mouse line DUhTP	Running-wheel exercise Treadmill exercise	Irisin (plasma and muscle) ↑ FNDC5 protein (muscle) —	Brenmoehl et al. (2014)
Mice	Running-wheel exercise	FNDC5 mRNA (hippocampal) ↑	Wrann et al. (2013)
C57BL/6 J mice	Treadmill exercise	Irisin (plasma) ↑ FNDC5 mRNA and protein (muscle) ↑	Park et al. (2022)
*Homo sapiens*	Acute exercise Chronic exercise	Serum irisin decreased after chronic exercise and increased after acute exercise	Norheim et al. (2014)
*Homo sapiens*	Aerobic exercise	Irisin (plasma) ↑	Kraemer et al. (2014)
*Homo sapiens*	Acute exercise Chronic exercise	Serum irisin was upregulated after acute exercise	Huh et al. (2012)
*Homo sapiens*	Endurance exercise Resistance exercise	Increased plasma irisin levels after resistance exercise	Tsuchiya et al. (2015)

## 5 Exercise-related irisin and depression

Several clinical trials have investigated the relationship between depression and exercise-produced irisin. A randomized trial by Bilek et al. showed that aerobic exercise elevated irisin serum levels and reduced depression and fatigue in patients with relapsing-remitting multiple sclerosis (Bilek et al., 2022). Tu et al. tracked patients with acute ischemic stroke for 6 months, finding that serum irisin levels were lower in patients with post-stroke depression (PSD) compared with patients who are not depressed. Irisin also outperformed other biomarkers, such as age and serotonin, in predicting PSD (Tu et al., 2018). Animal studies have found that irisin released during exercise improves depressive-like behaviors by increasing dopamine and norepinephrine levels (Yardimci et al., 2023). The antidepressant-like, pro-neurogenic and neuroprotective effects of exercise were associated with the FNDC5/Irisin pathway (Gruhn et al., 2021). Notably, aerobic exercise enhanced the activity of the PGC-1α/FNDC5/Irisin pathway and further increased BDNF levels in the hippocampus and prefrontal cortex (PFC) (Jo and Song, 2021). Therefore, exercise-induced irisin can exert antidepressant effects. Additionally, exogenous supplementation irisin exerts its antidepressant effects by regulating brain energy metabolism (Wang and Pan, 2016), promoting the expression of BDNF in various brain regions (Pignataro et al., 2022), reducing neuroinflammation, and decreasing the expression of epidermal growth factor receptors (EGFR) in mice (Hou et al., 2020), as shown in Table 2. Although studies suggest irisin has antidepressant effects, the exact mechanisms remain unclear. Understanding how irisin regulates depressive symptoms is crucial for developing therapeutic approaches to depression. Irisin has been shown to play key roles in regulating metabolism, reducing oxidative stress, decreasing inflammation, preventing apoptosis, and improving mitochondrial dysfunction (Askari et al., 2018; Jiang et al., 2021). Considering the involvement of irisin in multiple pathological processes, its role in depression may be complex. Therefore, irisin has great potential as an “exercise-mimicking” intervention in the treatment of depression.

**TABLE 2:** Experimental models of the effects of irisin on depression.

Model	Dose (route)	Behavioral test	Main findings	References
C57BL/6mice	100 μg/kg (I.P)	TST, FST, OFT	Irisin treatment decreased the immobility time in the TST and FST; BDNF and IGF-1gene expressions were significantly increased	Pignataro et al. (2022)
C57BL/6 mice	0.5–1 ng/mouse (I.C.V)	TST, FST, OFT	Irisin reduced the immobility time in the TST and FST; Hippocampal PGC-1αand BDNF mRNA levels increased after 6 h of irisin treatment	Siteneski et al. (2018)
propofol-treated mice	0.5 mg/kg (I.P)	TST, FST	Irisin decreased immobility time in the TST and FST in propofol-treated mice; Irisin could protect neurons, inhibit cytokine increase in astrocyte cultures exposed to propofol, and reduce epidermal growth factor receptor expression on cell surfaces	Hou et al. (2020)
CUS rats	100 ng/mL or higher	FST, SPT	Irisin increased the activity of mitochondrial complexes I, II, and IV, as well as the phosphorylation level of creatine kinase and glucose transport	Wang and Pan (2016)

Abbreviations: CUS, chronic unpredictable stress; ARS, acute restraint stress; TST, tail suspension test; OFT, open-field test; FST, forced swim test; SPT, saccharin preference test; I.P, intraperitoneal injection; I.C.V., intracerebroventricular.

## 6 The potential role of exercise-related irisin in depression

### 6.1 Irisin regulates energy metabolism

Impaired brain energy metabolism has gained increasing attention as a key factor in the pathogenesis of depression (Wu et al., 2016; Chan et al., 2019; Cao et al., 2013; Ernst et al., 2017). Combined proteomic and metabolomic analyses provide compelling evidence that patients with mood disorders exhibit abnormalities in energy and substance metabolism, which are linked to an elevated risk of developing depression (Wu et al., 2016; Qin et al., 2019; Liu et al., 2018). Disruptions in glucose, lipid, and amino acid metabolism have been widely reported in patients with major depressive disorder (MDD) (Qin et al., 2019; Liu et al., 2018; Chen et al., 2015).

Research increasingly shows that irisin plays a role in regulating lipid and glucose metabolism in skeletal muscle and adipose tissue (Boström et al., 2012; Luo et al., 2020; Xiong et al., 2015; Moreno-Navarrete et al., 2013). In obese mice, FNDC5/irisin improves insulin resistance, corrects glucose and lipid metabolic abnormalities, and enhances lipolysis via the cAMP-PKA-HSL/perilipin pathway (Xiong et al., 2015). Irisin promotes the expression of UCP1 and the conversion of WAT to BAT, which increases thermogenesis and energy expenditure to improve metabolism (Boström et al., 2012; Zhang et al., 2014). This effect may be mediated by irisin-induced activation of the p38 MAPK and extracellular signal-related kinase (ERK) signaling pathways (Zhang et al., 2014). Additionally, central irisin injection has been shown to considerably increase oxygen consumption and carbon dioxide production in rats, along with heat generation (Zhang et al., 2015), suggesting that irisin can markedly enhance metabolic activity and may be an effective treatment for metabolic diseases. Wang et al. found that irisin treatment improved prefrontal cortical energy metabolism in CUS rats, as shown by increased activity of mitochondrial complexes I, II, and IV, resulting in improved depression-like behaviors (Wang and Pan, 2016). Impaired brain glucose metabolism and insulin signaling are key pathological features of depression and contribute to its onset and progression (Ernst et al., 2017; Kleinridders et al., 2015; Kimbrell et al., 2002). Some studies have reported that patients with depression exhibit higher-than-normal blood glucose levels, and elevated glucose levels have been associated with symptoms of fatigue in patients with mild depression. Kimbrell et al. (2002) discovered that patients with monophasic depression had lower regional cerebral glucose metabolism in the dorsal prefrontal and anterior cingulate cortices compared with healthy controls, with glucose metabolism negatively correlated with depression severity. Numerous studies have demonstrated that irisin’s positive effects on insulin sensitivity and glucose metabolism, suggesting that maintaining glucose homeostasis may be another mechanism through which irisin regulates metabolism. Plasma levels of irisin are linked to T2D and obesity, with circulating irisin levels inversely correlated negatively with insulin resistance (Moreno-Navarrete et al., 2013). Serum irisin levels are reduced in patients with T2D (Choi et al., 2013). Irisin enhances fatty acid oxidation and glucose consumption in T2D by modulating the AMPK signaling pathway and reducing the expression of the gluconeogenic enzymes PEPCK and G6Pase in the liver (Xin et al., 2016). Furthermore, irisin activates p38 MAPK in an AMPK-dependent manner, inhibiting or knocking p38 MAPK blocks irisin-induced glucose uptake (Lee et al., 2015). Irisin also increases the expression of β-trophin, a hormone recently discovered to promote pancreatic β-cell proliferation and improve glucose tolerance (Zhang et al., 2014). Interestingly, increased endogenous ATP release from astrocytes has been shown to have antidepressant-like effects in mouse models of depression, Suggesting a physiologic link between ATP release from astrocytes and MDD (Cao et al., 2013). Irisin treatment markedly boosts levels of key glucose metabolism enzymes, including type I and type II hexokinase, and glucose transporters GLUT-4 in astrocyte membranes (Wang and Pan, 2016). Based on these findings, we hypothesize that irisin may serve as a regulator of energy metabolism, particularly glucose metabolism and insulin activity, in patients with depression.

### 6.2 Irisin regulates the expression of BDNF

BDNF is highly expressed in the central nervous system and plays a critical role in synaptic function, neurotransmission, and neurogenesis (Numakawa et al., 2018). A substantial amount of clinical and experimental data suggests that BDNF is integral to the pathophysiology of depression. Reduced BDNF production and signaling have been observed in *postmortem* brain tissue of patients with depression (Sheldrick et al., 2017). Antidepressant treatments have been shown to increase BDNF synthesis and signaling, with direct BDNF infusion into the hippocampus potentially providing antidepressant effects (Zavvari and Nahavandi, 2020). However, selective loss of BDNF in the DG has been found to reduce antidepressant efficacy (Björkholm and Monteggia, 2016).

Exercise induces hippocampal BDNF expression via the PGC-1α/FNDC5 pathway (Wrann et al., 2013). Moreover, intraperitoneal administration of recombinant irisin increases PGC-1α, FNDC5, and BDNF mRNA levels in the hippocampus (Kim and Leem, 2019). Peripheral delivery of FNDC5 to the liver via adenoviral vectors also elevates blood irisin levels, promoting BDNF and other neuroprotective gene expressions in the hippocampus (Wrann et al., 2013). Further research by Islam et al. demonstrated that exercise-induced irisin can cross the BBB via peripheral transport, inducing BDNF expression in the CNS (Islam et al., 2021). These findings suggest that FNDC5/irisin can cross the BBB and regulate gene expression in the brain. Huang et al. conducted a study on diabetic rats, dividing them into four groups: control, model, irisin, and irisin-shRNA. They found that irisin and BDNF levels were significantly lower in the model and irisin-shRNA groups but significantly higher in the irisin group, indicating that irisin promotes BDNF expression (Huang et al., 2019). Wrann et al. confirmed similar results when they inhibited FNDC5/irisin expression in cortical neurons using siRNA, resulting in decreased BDNF expression (Wrann et al., 2013). Further studies revealed that recombinant irisin activated the cAMP/PKA/CREB pathway in human cortical slices and increased cAMP and pCREB in mouse hippocampal slices (Lourenco et al., 2019), a pathway crucial for BDNF synthesis. It is well-established that activating the cAMP/PKA/CREB/BDNF signaling pathway and upregulating BDNF expression have antidepressant effects (Wu et al., 2021; Cai et al., 2022). Siteneski et al. investigated the impact of central irisin administration on BDNF mRNA expression and protein levels, finding that irisin reduced BDNF mRNA expression in the hippocampus after 1 h but increased it after 6 h (Siteneski et al., 2018). BDNF mRNA expression paralleled FNDC5 mRNA expression (Siteneski et al., 2018), consistent with FNDC5 being a positive regulator of BDNF expression (Wrann et al., 2013). Additionally, genetic polymorphisms in BDNF (BDNF Met/Met) prevented exercise from producing antidepressant effects and elevating BDNF and FNDC5 mRNA levels in the DG of the hippocampus (Ieraci et al., 2016). Given that BDNF expression may help mitigate the onset and progression of depression, activating the FNDC5/irisin-BDNF axis in the brain may offer a promising therapeutic approach for depression.

### 6.3 Irisin promotes neurogenesis and synaptic plasticity

Depression is associated with reduced adult neurogenesis in the hippocampus, a reduction that can be alleviated or restored through chronic antidepressant treatment (Wang et al., 2008; Tunc-Ozcan et al., 2019). Brain imaging studies have shown decreased volume in cortical and limbic regions, particularly in the PFC and hippocampus, in patients with MDD. This volume reduction is more pronounced in patients with multiple episodes, recurrent relapses, and longer disease duration (Lorenzetti et al., 2009). Cotter et al. (2002) analyzed *postmortem* brain tissue from patients with MDD and found diminished glial cell density and neuronal volume in the dorsolateral PFC. Furthermore, reduced synaptic plasticity may contribute to impaired functioning and the loss of the ability to regulate mood and cognition in patients with depression (Duman et al., 2019). Kang et al. discovered that synaptic function-related genes (CALM2, SYN1, RAB3A, RAB4B, and TUBB4) were less expressed in the PFC of patients with MDD PFC, leading to a decrease in the number of synapses (Kang et al., 2012). In a repeated restraint stress model, stress decreased the number of dendritic spines in the medial PFC of rats, indicating not only a reduction in the total number of axons but also potential impairment of neuroplasticity in these regions (Radley et al., 2006).

FNDC5 may play a role in neuronal differentiation and genesis (Hashemi et al., 2013; Forouzanfar et al., 2015; Ebadi et al., 2021). A previous study by Hashemi et al. found that FDNC5 expression increased during neurogenesis in retinoic acid-treated mouse embryonic stem cells (mESCs) (Ostadsharif et al., 2011). Subsequent experiments revealed that knockdown of FNDC5 in neuronal precursor cells inhibited the differentiation of mouse mESCs into neurons and the maturation of astrocytes (Hashemi et al., 2013). Ebadi et al. further demonstrated that mRNA and protein levels of BDNF and its receptors, tyrosine kinase (Trk) and p75, decreased following FDNC5 knockdown, concluding that FNDC5 knockdown inhibits neuronal differentiation by affecting neurotrophic factor expression (Ebadi et al., 2021). In contrast, overexpression of FNDC5 promotes the expression of neuronal precursor markers Sox1 and Pax6, mature neuronal markers such as Neurocan, and the astrocytic marker GFAP, likely due to enhanced BDNF expression following FNDC5 overexpression (Forouzanfar et al., 2015). This underscores the significance of FNDC5 in neuronal differentiation.

The motor-FNDC5/irisin-BDNF axis has been shown to enhance neuroplasticity, including neuronal growth, survival, and synaptic stabilization. Exercise increases plasma irisin levels, elevates hippocampal BDNF expression and BrdU-positive cells, and alleviates cognitive decline and depressive states associated with physical inactivity (Park et al., 2022). Low to moderate-intensity exercise lasting 4 weeks improved depression-like behavior in mice and increased neuronal proliferation, differentiation, and survival in the hippocampus, correlated with elevated hippocampal FNDC5/irisin levels post-exercise (Siteneski et al., 2020). Intraperitoneal administration of recombinant irisin activates the hippocampal PGC-1α/FNDC5/BDNF signaling pathway, enhancing dendritic length and spine density in the CA1 and CA3 regions but not in the DG (Kim and Leem, 2019). Exercise-mediated irisin also plays a crucial role in improving cognitive function, with plasma irisin levels positively correlated with hippocampal BDNF concentrations and cell proliferation (Park et al., 2022). In Alzheimer’s disease models, irisin can activate the cAMP/PKA/CREB signaling pathway in the brain, increasing BDNF expression, promoting neurogenesis and synaptogenesis, and enhancing synaptic plasticity, thereby improving cognitive function (Lourenco et al., 2019). Thus far, both exercise and BDNF have been associated with increased neurogenesis. Interestingly, BDNF is not the only factor influencing hippocampal neurogenesis; neurogenesis-related STAT3 signaling has also been shown to affect proliferation (Moon et al., 2013a; Yang et al., 2015). Moon et al. explored whether irisin could activate STAT3/AMPK/ERK signaling in mouse HT19-7 HN cells to influence hippocampal neurogenesis. They found that pharmacological concentrations of irisin (50–100 nmol/L), but not physiological concentrations (5–10 nmol/L), increased the proliferation of mouse hippocampal neuronal cells via the STAT3 signaling pathway. Notably, neither concentration affected neurogenesis in hippocampal neuronal cells nor activated AMPK or ERK signaling pathways (Moon et al., 2013b). There may be other signaling pathways activated by irisin that have yet to be reported, indicating a need for further research to elucidate the regulation of irisin levels and its physiological effects on hippocampal neurogenesis. Taken together, this discussion suggests that FNDC5/irisin plays a role in promoting neurogenesis and enhancing synaptic plasticity, providing a novel approach to improving depression-like behavior.

### 6.4 Irisin inhibits neuroinflammation

Neuroinflammation is known to play a crucial role in the pathogenesis of depression. Pro-inflammatory cytokine levels, such as IL-6 and tumor necrosis factor α (TNF-α), are elevated in the serum and cerebrospinal fluid of patients with depression (Haapakoski et al., 2015). Elevated mRNA and protein levels of interleukin-1β (IL-1β), IL-6, and TNF-α have also been observed in the PFC of patients with depression who died by suicide (Pandey et al., 2018). Studies involving animal models indicate that depressive-like behavior is linked to increase inflammation markers in peripheral and MDD-associated brain regions (Lu et al., 2017). Administration of lipopolysaccharide (LPS), which triggers an immune and inflammatory response, has been shown to induce depressive-like behaviors in rodents (Franklin et al., 2018).

In recent years, the anti-inflammatory, anti-apoptotic, and antioxidant properties of irisin in neurological diseases have garnered considerable attention (Askari et al., 2018). Although no definitive mechanism has been established regarding how irisin suppresses inflammation, it is hypothesized that it may inhibit inflammatory signal transduction systems and/or the nucleotide-binding oligomerization domain-like receptor containing pyrin domain 3 (NLRP3) inflammasome. The toll-like receptor 4 (TLR4)/Myeloid differentiation primary response gene 8 (MyD88)/NF-κB pathway is a classical inflammatory signaling pathway involved in the development of depression (Xiao et al., 2022). A study by Mazur-Bialy et al. found that high concentrations of irisin (50, 100 nM) attenuated LPS-stimulated inflammatory activation of macrophages and reduced the release of pro-inflammatory cytokines by inhibiting the downstream TLR4/MyD88 pathway and NF-κB phosphorylation, which is associated with irisin’s effects on MAPK phosphorylation (Mazur-Bialy et al., 2017). Yu et al. (2020) constructed a middle cerebral artery occlusion (MCAO) model to study the role of irisin in cerebral ischemia-reperfusion injury, obtaining similar results; irisin inhibited neuroinflammatory responses and reduced neuronal injury by downregulating TLR4/MyD88 and inhibiting NF-κB activation. In another study, irisin treatment inhibited the activation of Iba-1 microglia, infiltration of monocytes, oxidative stress, and the expression of inflammatory factors (TNF-α and IL-6) in mice with MCAO (Li et al., 2017), a finding confirmed in P12 neuronal cells. Moreover, pretreatment with neutralizing antibodies to irisin significantly attenuated the reduction in neuroinflammation observed following physical exercise (Li et al., 2017). Irisin suppressed the release of pro-inflammatory cytokines such as IL-1β, IL-6, TNF-α, and COX-2 in a rat spinal cord injury model through the AMPK-NF-κB pathway (Jiang et al., 2020), with these findings further verified in an LPS-induced P12 cell injury model (Jiang et al., 2020). In a brain hemorrhage model, irisin inhibited microglia/macrophage pro-inflammatory polarization, reduced neutrophil infiltration, and downregulated the expression of pro-inflammatory cytokines TNF-α and IL-1β by upregulating the integrin αVβ5/AMPK signaling pathway (Wang et al., 2022). An *in vitro* assay investigating whether irisin protects neurons from Aβ-induced cellular damage showed that irisin attenuates the release of IL-6 and IL-1β and reduces COX-2 expression levels in astrocytes. Finally, irisin can reduce NF-κB activation in astrocytes exposed to Aβ by blocking phosphorylation and loss of I κ Bα (Wang et al., 2018). Another study indicated that irisin could attenuate inflammation induced by oxygen-glucose deprivation by inhibiting reactive oxygen species (ROS) and the NLRP3 inflammatory signaling pathway (Peng et al., 2017).

Irisin plays a significant role in macrophage polarization and its anti-inflammatory function. Macrophages are classified as either traditionally activated (M1-type) or alternatively activated (M2-type), which have opposing roles in inflammation (Duan et al., 2022; Kawanishi et al., 2010; Van den Bossche et al., 2016). M1-type macrophages produce pro-inflammatory cytokines, including TNF-α, IL-6, and IL-1β; in contrast, M2-type macrophages secrete anti-inflammatory cytokines, such as IL-10 (Van den Bossche et al., 2016). Xiong et al. (2018) demonstrated that exogenous FNDC5 and FNDC5 overexpression inhibited LPS-induced M1 macrophage polarization and inflammatory cytokine production via the AMPK pathway. Previous studies have shown that irisin treatment can inhibit the expression of pro-inflammatory cytokines, reduce macrophage migration, and induce a phenotypic switch in macrophages from an M1 to an M2 state (Dong et al., 2016). Notably, irisin-mediated BDNF upregulation has the potential to reduce neuroinflammation by inhibiting the synthesis of NF-κB and pro-inflammatory cytokines IL-6 and IL-1β via activation of the ERK-CREB pathway through its receptor TrkB (Madhu et al., 2022). In conclusion, irisin reduces neuroinflammation and decreases the production of inflammatory factors in the brain. These effects of irisin in inflammatory conditions suggest potential therapeutic applications for irisin in the context of depression-related neuroinflammation.

### 6.5 Irisin blocks oxidative stress to prevent neuronal damage

Dysregulated redox homeostasis is implicated in the pathophysiology of depression (Pandya et al., 2013; Zuo et al., 2022; Bhatt et al., 2020). Oxidative stress can initiate or exacerbate several pathogenic processes associated with depression, including iron death, neuroinflammation, impaired autophagy, and mitochondrial dysfunction (Zuo et al., 2022). Meta-analyses have shown impaired antioxidant capacity and increased levels of oxidative damage products in patients with depression. Notably, antioxidant levels have been found to increase with the use of antidepressants (Liu et al., 2015; Palta et al., 2014). Therefore, inhibiting oxidative stress may improve depressive symptoms, as some antioxidants exhibit potential antidepressant efficacy (Eren et al., 2007). Oxidative stress refers to an imbalance between the generation of ROS and antioxidant defenses (Betteridge, 2000). High levels of ROS can damage proteins and DNA while promoting the release of inflammatory mediators, ultimately leading to cell death and apoptosis (Bhatt et al., 2020; Juszczyk et al., 2021). Furthermore, uncoupling protein 2 (UCP2), expressed in the central nervous system, has demonstrated strong neuroprotective effects (Wang et al., 2014; Hoang et al., 2012). UCP2 reduces mitochondria-mediated ROS production through uncoupling, increases ATP levels, mitigates mitochondrial damage caused by free radicals, and assists neural cells in utilizing energy derived from free radicals. UCP2 deficiency has been shown to exacerbate depression-like behavior and promote mitochondrial damage and ROS production in astrocytes in a chronic mild stress model (Du et al., 2016).

Irisin has been shown to protect against neuronal damage caused by oxidative stress in various neurological disease models. In the MCAO model, irisin considerably reduced levels of nitrotyrosine, superoxide anion, and 4-hydroxynonenal in peri-infarct brain tissue by activating AKT and ERK1/2 signaling pathways. It also inhibited the secretion of pro-inflammatory factors and alleviated ischemia-induced neuronal damage (Li et al., 2017). In a mouse model of oxygen-glucose deprivation (OGD), irisin mitigated OGD-induced neuronal damage by blocking the ROS-NLRP3 inflammatory signaling pathway and reducing ROS and malondialdehyde production to inhibit oxidative stress (Peng et al., 2017). In rat models of kainic acid-induced status epilepticus and chronic spontaneous epilepsy, exogenous irisin considerably enhanced the expression of BDNF and UCP2 while reducing levels of neuronal damage and mitochondrial oxidative stress (Yu et al., 2022; Cheng et al., 2021). In a mouse model of traumatic brain injury, exogenous irisin alleviated inflammatory responses and oxidative stress by inducing UCP2 expression in neuronal mitochondrial membranes, leading to reduced mitochondrial damage and decreased ROS production and malondialdehyde content (Guo et al., 2021). Consequently, irisin is a critical regulator of oxidative stress and a potential therapeutic agent for depression.

## 7 Conclusion

**FIGURE 3:**
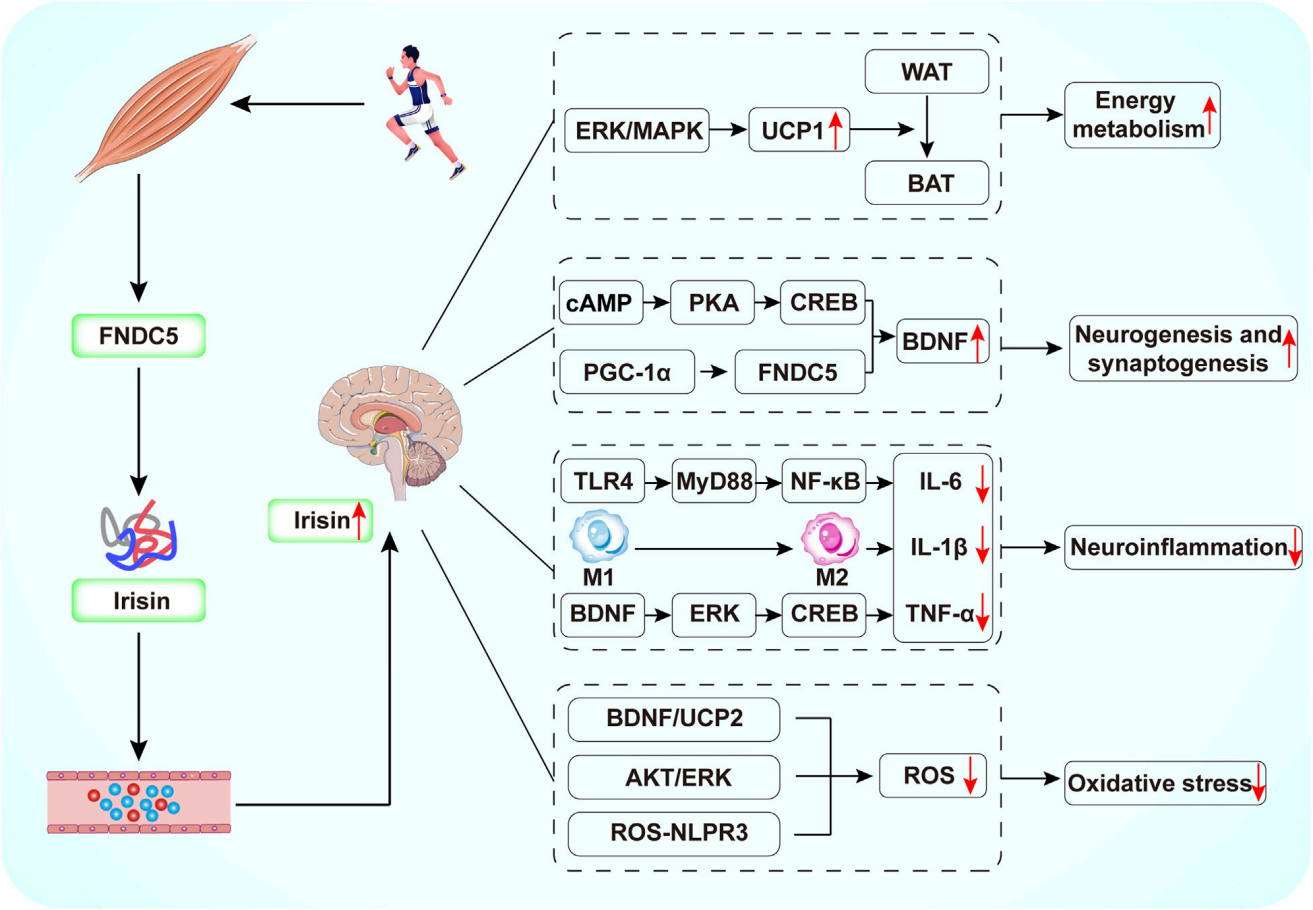
Schematic diagram of the potential mechanisms by which exercise-mediated release of irisin exerts antidepressant effects. Exercise induces FNDC5 expression in skeletal muscle. The FNDC5 protein is subsequently cleaved to irisin, which enters the brain by crossing the BBB. Irisin can increase BDNF production by activating cAMP/PKA/CREB, PGC-1/FNDC5 signaling. Irisin inhibits NF-κB phosphorylation through downregulation of TLR4/MyD88 pathway, induces the conversion of M1-type macrophages to M2-type macrophages, and activates the BDNF/ERK/CREB pathway, thereby reducing the production of pro-inflammatory cytokines. In addition, irisin reduces ROS production by activating the BDNF/UCP2 and AKT/ERK pathways and by inhibiting the ROS-NLPR3 inflammatory signaling pathway.

Compared to antidepressants, exercise is effective in improving depressive symptoms without the toxic side effects associated with medications. However, for patients with depression who are more severely ill and unable to engage in regular physical activity, irisin, as an exercise mimetic, has great potential in the treatment of depression. Research suggests that exercise-induced FNDC5/irisin plays a key role in connecting muscle and brain function. This review highlights the potential benefits of irisin in depression, presenting five key theories regarding its role in the depressed brain (Figure 3). First, irisin can improve metabolic dysfunction in the brain by promoting energy expenditure and regulating glucose and lipid metabolism. Second, FNDC5/irisin can increase hippocampal BDNF expression, promoting neurogenesis and synaptic plasticity. Third, irisin can attenuate neuroinflammatory responses. Fourth, it inhibits oxidative stress, thus preventing neuronal damage. Additionally, irisin levels may serve as an important biomarker for the diagnosis or treatment of depression. Therefore, further in-depth animal and clinical studies on the mechanisms by which irisin alleviates depression could facilitate the development and testing of new treatments, reduce the incidence of depression, and provide benefits for patients unable to exercise due to physical limitations.

Prospectively, several unexplored areas deserve more intensive research. Exercise can significantly alter the structure, composition, and abundance of the intestinal microbiota, thereby affecting overall health. In recent years, the complex interrelationship between exercise, intestinal microbiota and depression has received much attention. However, conclusive studies on whether irisin can treat depression by modulating the intestinal microbiota have not yet been conducted. Therefore, this area of research deserves more studies. Furthermore, the application of irisin in the clinical setting is challenging due to its short half-life *in vivo*. Fortunately, there are several approaches to extend the lifespan of protein drugs, such as Fc fusion proteins and coupling with albumin (Glaesner et al., 2010; Sleep et al., 2013). Additionally, the emergence of nanotechnology-based drug delivery strategies, like hydrogels, offers potential solutions, promising to prolong the therapeutic effect and reduce the frequency of administration (Tsou et al., 2019). Future research should aim to enhance the stability of irisin using these innovative modifications to optimize therapeutic outcomes and expand its clinical potential. It should be emphasized that irisin is still in its infancy as a potential therapeutic strategy for depression. There is a lack of data from large-scale clinical trials to validate its safety and efficacy, as well as to clarify its dosage range and time window for clinical application. Therefore, further research and validation are needed in the future to deeply explore the potential value of irisin in the treatment of depression.
